# Right from the start: protocol for a pilot study for a randomised trial of the New Baby Programme for improving outcomes for children born to socially vulnerable mothers

**DOI:** 10.1186/s40814-018-0235-2

**Published:** 2018-02-02

**Authors:** Geraldine Macdonald, Fiona Alderdice, Mike Clarke, Oliver Perra, Fiona Lynn, Theresa McShane, Sharon Millen

**Affiliations:** 1 School for Policy Studies, University of Bristol, 8 Priory Road, Bristol, BS8 4BQ UK; 2 National Perinatal Epidemiology Unit, Nuffield Department of Population Health, University of Oxford, Old Road Campus, Oxford, OX3 7LF UK; 3 School of Nursing and Midwifery, Queen’s University Belfast, Health Sciences Building, 97 Lisburn Road, Belfast, BT9 7BL Northern Ireland, UK; 4 School of Medicine, Dentistry and Biomedical Sciences, Queen’s University Belfast, Health Sciences Building, 97 Lisburn Road, Belfast, BT9 7BL Northern Ireland, UK

**Keywords:** Early intervention, Attachment, Child maltreatment, Health visiting, Home visiting, Social complexity, Pregnancy

## Abstract

**Background:**

Children born to mothers who experience social complexity (e.g. substance misuse, intimate partner violence, mental ill health, a history of maltreatment) are at increased risk for a range of adverse outcomes at birth and during development. Home visiting programmes have been advocated as a strategy for improving outcomes for disadvantaged mothers and children, such as the Nurse-Family Partnership for young, socially disadvantaged first-time mothers. However, no evidence-based programme is available for multiparous women or older first-time mothers. The New Baby Programme was developed in Northern Ireland. It augments the universal health visiting service available in the UK with a content designed to promote maternal health and well-being in pregnancy, maximise secure attachments of children and parents and enhance sensitive parenting and infant cognitive development.

**Methods/Design:**

This pilot study is designed to investigate whether it is possible to recruit and retain socially vulnerable mothers in a randomised trial that compares the effects of the New Baby Programme with standard antenatal and postnatal care. Feasibility issues include the referral/recruitment pathway (including inclusion and exclusion criteria), the consent and randomisation, the ability to maintain researcher blinding, the acceptability of the intervention to participants, and the feasibility and acceptability of the outcome measures. The results of the study will inform a definitive phase-3 RCT.

**Discussion:**

Trials of complex social interventions often encounter challenges that lead to the trial being abandoned (e.g. because of problems in recruitment) or present considerable analytic challenges relating to dropout, attrition and bias. This pilot study aims to maximise the chances of successful implementation.

**Trial registration:**

ISRCTN35456296 retrospectively registered

## Background

Social circumstances may adversely affect the outcomes of pregnancy, both for women and their baby [1]. *Saving Mothers Lives* documents the increased risk of death during or after pregnancy of socially disadvantaged or excluded women in England and Wales [2]. Of the women who died, those with socially complex lives were less likely to contact maternity services early or to stay in regular contact. Compared with women who booked before 20 weeks into their pregnancy, those who booked late or missed more than four regular appointments were more likely to be black African or Caribbean, experiencing domestic abuse, misusing substances, known to child protection services, or unemployed [3].

*Perinatal Mortality* (2009) highlighted the disparity in rates of stillbirth and neonatal death between white women and those from ethnic minority groups in the UK [4]. Unemployment, socioeconomic deprivation, ethnicity (African and African Caribbean, Indian and first-generation migrants from Pakistan) and later antenatal booking appointments (past 13 weeks) are all associated with increased risk of stillbirth [2, 5, 6]. Complex social factors may enhance stress, now recognised as deleterious to the health of both mother and child [7, 8].

Children born to mothers whose circumstances feature social complexity are at increased risk of a range of adverse outcomes, including birth outcomes (e.g. low birth weight, preterm delivery), health problems in infancy and impaired development. Some of these are associated with poor maternal health during pregnancy and/or behaviours that can adversely impact on the developing child. Key concerns include smoking [9], substance misuse [10], intimate partner violence [11], mental ill health [12, 13], socio-economic deprivation and a history of maltreatment [14, 15].

Despite a small reduction in inequality of pregnancy outcomes between the most deprived and better off women in the UK population [16, 17], the problem remains a significant one, with serious implications for the many children living with parents coping with social complexities.

### Home visiting programmes

Home visiting programmes have been advocated as a strategy for improving the health of disadvantaged children, and in 2006, the prime minister, Tony Blair, identified home visiting programmes as an ‘early intervention’ that could help combat social exclusion [18]. Unlike universal health visiting, home visiting programmes often start antenatally and may continue for 2 years postpartum. Typically, they involve structured visits by those experienced in child health and development. Some programmes use professionals as home visitors (on the grounds that parents value ‘expert’ and ‘confidential’ advice and support), whilst others use trained and supervised lay visitors (on the grounds that those who have ‘been there’ are better able to engage those who are experiencing difficulty). Home visiting programmes may target maternal smoking, poor attachment, poor nutrition, the under-stimulation of children, lack of social support and a range of factors known to increase risk for maltreatment [19–23]. They may seek to improve the economic status of families by helping mothers complete their education, secure employment and plan for any future pregnancies [24].

Home visiting programmes are often designed specifically to engage women who are traditionally found ‘difficult to engage’ by routine services. Intervention takes place within the home because this is where most parenting takes place. As well as providing natural opportunities to model, practice, and reinforce good parenting, home visiting maximises the chances of parental engagement. Reduced caseloads enable staff to visit more frequently to be available between scheduled visits and to provide a wider range of support services, such as help with practical tasks (e.g. arranging and keeping appointments with other agencies, negotiating with welfare agencies, general advocacy). Staff usually receive additional training in order to deliver these programmes. This may include enhancing their understanding of the wider context of parenting (e.g. how childhood maltreatment or mental health problems can influence parenting) and developing skills associated with enhancing parental engagement (e.g. motivational interviewing).

### Nurse-Family Partnership (NFP)

NFP is based on a number of psychological theories, such as theories of cognitive development [25], attachment theory [26] and social learning theory [27, 28], organised within an ecological framework. NFP operates on the understanding that successful programmes must be able to make significant impacts on ‘the enduring environment in which the family is functioning’ [29] (p.12). The programme assumes that new parents are committed to the well-being of their child and that most will act in ways they deem to be appropriate during pregnancy and subsequent years. Failures of parenting are therefore assumed to reflect a lack of knowledge about what their children need or to arise from other factors such as mental illness or life stressors. NFP is targeted solely at first-time mothers on the basis that it is at this point that mothers are maximally motivated to engage in behaviour change to promote the well-being of their child.

NFP has been evaluated in three randomised trials, in three locations in the USA. Each sample was different, but all were socially disadvantaged. Outcomes and measures differ across the three trials, and the programme developers were responsible for these three evaluations (and have been involved in the small number of more independent evaluations). Generally, the three trials were well designed and implemented and indicate positive impacts in a range of maternal and child outcomes. However, recent trials have found no evidence of impact in the UK [30, 31]. NFP has been commissioned for use within the UK, where it is known as the Family-Nurse Partnership (FNP). As a licenced programme, the cost is considerable, and there is currently no comparable provision for the significant numbers of older and/or multiparous mothers presenting with complex social factors. The New Baby Programme (NBP) aims to bridge this gap.

### The New Baby Programme

The NBP was developed within the South Eastern Health and Social Care Trust in Northern Ireland. It aims to promote maternal health and well-being in pregnancy, maximise secure attachments and enhance sensitive parenting and infant cognitive development. In setting these aims, the programme drew on evidence concerning the importance of maternal health and well-being in pregnancy for optimal in utero development (e.g. [32–36]) and the importance of secure attachments [37–40] and ensuring healthy social and emotional development in the early years [8, 41, 42].

**Fig. 1:**
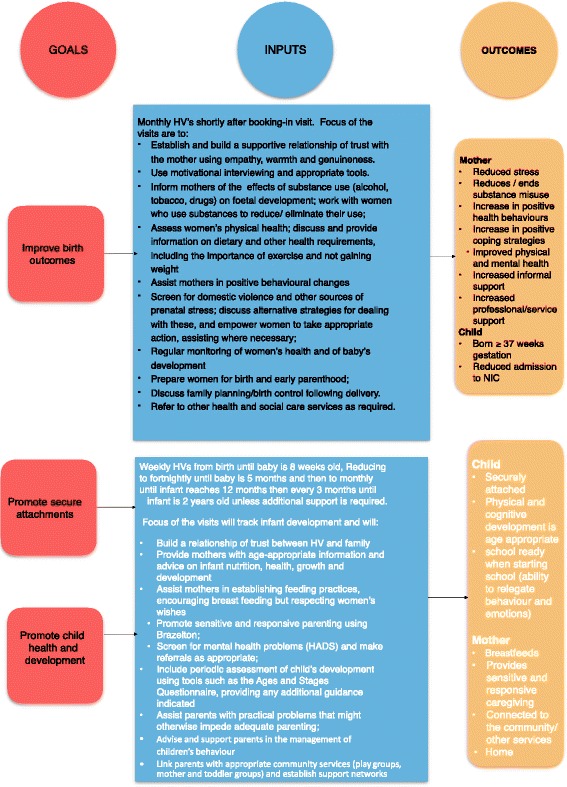
Logic model

Exposure to the same stressor does not necessarily lead to the same outcome for all who experience it (multifinality). Similarly, there are many paths to the same type of outcome (equifinality). Consequently, interventions such as home visiting are necessarily broad-brush in some respects. Promoting optimal prenatal health will require a different strategy for a mother who does not smoke or drink than one who does; it will look different for a mother who is homeless than one who is not, and so on. The logic model underpinning the NBP reflects this complexity (see Fig. 1). The programme seeks to combine a core curriculum of evidence-based advice, information and interventions with the opportunities and flexibility afforded to health visitors with small caseloads, additional training and specialist supervision.

A service evaluation of the NBP, conducted in 2013, identified the programme as a promising intervention, well received by staff and parents, but needing further work to describe and manualise the intervention and to more tightly specify the training and supervisory needs of those delivering it. This work has been undertaken. The Public Health Agency in Northern Ireland wishes ultimately to secure an independent evaluation of the programme using a randomised controlled trial (RCT). The primary objective of an RCT would be to determine whether the NBP, compared with routine antenatal and postnatal care, can improve infant attachment and maternal sensitivity among pregnant women with complex social factors and the quality of maternal-child relationships. The secondary objectives would be to determine whether the NBP can improve maternal mental health and outcomes for children, at birth and during the first year of life.

### Aims of the study

This pilot study is designed to test the feasibility of an RCT to investigate the effectiveness of the New Baby Programme for pregnant women of any age presenting with socially complex circumstances. It will determine the recruitment and retention rates required, the process of randomisation, the procedures for masking allocation status from the research team and the acceptability of both the intervention and participation in the study. Particular attention will be paid to the feasibility and acceptability of the outcome measures used, as these require video-taping of mothers and babies, one of which will entail attendance at a clinic.

### Research questions

The research questions for the study are as follows:

- Is the study design feasible? Is it possible to recruit and retain women with socially complex circumstances into an RCT and collect data at 20 weeks of gestation and then when infants are 2, 6 and 12 months old?
- Is the intervention acceptable to participants and do they engage with it?
- Are the proposed outcome measures acceptable to participants and are they willing to collaborate in providing the required data?
- How many participants will be needed for a sufficiently powered RCT, and what time frame will be needed for recruitment?

#### Primary outcomes

1. Recruitment as a percentage of eligible participants

2. Retention assessed by percentage of participants randomised for whom data are available at baseline and 2, 6 and 12 months postpartum

#### Secondary outcomes

3. Acceptability of trial randomisation and data collection

4. Ability to maintain researcher blinding

5. Acceptability of the intervention and comparator

6. Acceptability of the outcome measures

## Methods/Design

### Design

This study comprises a pilot randomised trial in which eligible women are allocated to either the New Baby Programme or standard care. The social circumstances of the population targeted are such that recruitment and retention may be particularly challenging, so the study includes a qualitative component designed to provide information about the acceptability of the trial processes to participants and other key stakeholders (e.g. midwives, social care professionals, and GPs).

### Participants

In total, we aim to recruit 50 women with socially complex circumstances presenting for antenatal care at the Ulster, Ards and Bangor Hospitals (County Down) in the South Eastern Health and Social Care Trust in Northern Ireland. Twenty-five women will be randomised to receive the New Baby Programme. The remaining 25 women will receive Universal Core Programme recommended in the Healthy Child Programme through midwifery and health visiting services. This is the routine package of care that will be offered to those who do not wish to take part in the randomised trial. We aim to recruit the target sample of 50 participants over a 10-month period (Fig. 1).

#### Inclusion criteria

Participants will be eligible for the study if they are first time pregnant women over 19 years of age or multiparous women of any age (with a gestational age less than 18 weeks) presenting with one or more of the following: (i) social isolation/low family support/father in prison; (ii) intimate partner violence; (iii) substance misuse; (iv) maternal stress or history of mental ill health; (v) current involvement with social services or probation; (vi) history of care or a care leaver; (vii) and abnormal reaction to pregnancy.

#### Exclusion criteria

Women will be excluded if they do not meet any of the inclusion criteria of risks factors for social complexity and/or if they are under the age of 12 years or they are unable to speak sufficient English to take part in the intervention arm (identified as difficulty reading patient information sheets).

Midwives will use a screening tool (in the form of a vulnerability checklist) at the 8–18 weeks booking-in visit to determine women’s eligibility for the study.

### Programme delivery

#### Intervention

The New Baby Programme will be delivered by experienced health visitors specifically recruited for the study. They will receive an induction programme designed to familiarise them with the programme and the particular challenges of working with pregnant women whose social circumstances are complex. Training will be delivered by a specialist trainer together with a health visitor experienced in delivering the programme and working with this group of women.

#### Comparator

The Universal Core Programme (UCP) will be delivered by health visitors working in the same area. The UCP is itself a significant service provided to expectant mothers and their children, in the UK. It includes screening tests, immunizations, developmental reviews, information and guidance for parents. There is a strong emphasis on pregnancy and the first year of life [43]. Referral onwards to a range of other services, including specialist services (such as drug and alcohol services) is included in the Healthy Child Programme.

### Recruitment and informed consent

Recruitment will be undertaken via midwives at the three participating hospitals. Eligibility for the study will be determined by midwives who will use a screening tool (in the form of a vulnerability checklist) at the 8–18 weeks booking-in visit. They will briefly inform all eligible women about the study and invite them to take part. They will also provide eligible mothers with accessible, written information and inform them that a specialist nurse may telephone them to provide more details of the study. A senior nurse (a trust manager) from the Ulster Hospital will then telephone mothers to confirm eligibility and establish if the pregnancy is still viable. Women who are not eligible will be thanked for their time. The specialist nurse will ask eligible mothers if they would be interested in taking part and, if so, whether they are willing to be contacted by a named researcher (details of all members of the research team will be included on the information sheet). If the eligible mother is still interested in taking part and is willing for her contact details to be passed to the trial manager, then one of the specialist nurses will contact the research team.

One week following their booking-in visit, a researcher will contact the eligible mother to confirm if she is still willing to take part and, if so, to arrange a suitable time to visit her at her home. During this visit, the expectant mother will be asked to sign a consent form and baseline data will be collected.

During the baseline visit, the researcher will explain the study, including the possibility of any woman receiving the New Baby Programme (NBP) or Management as Usual (MAU), and how this will be determined (i.e. randomisation by a third party not directly involved in the study). In part to recognise their contribution to the study and to encourage retention, participants will be given a £20 gift voucher at completion of each data collection (baseline and 2, 6 and 12 months postpartum) and on completion of the Strange Situation Procedure [55].

### Randomisation

Randomisation is overseen by the School of Nursing and Midwifery (SNM) at Queen’s University, Belfast. It will use a central computer randomisation service (TENALEA) which employs a simple randomisation design in order to maintain complete randomness of the assignment of participants to either the intervention or comparator group.

Having secured written consent from a participant, the researcher will, on return to the office, enter the necessary details into TENALEA. The researcher will be provided with a unique study identifier for the participant but (in order to maintain the blinding of the researcher) not the random allocation. The random allocation will be conveyed by email from TENALEA to a designated officer in the School of Nursing and Midwifery for relaying to the relevant staff in the South Eastern Trust.

### Intervention outcome measures

An overview of the measures and the timetabling of data collection can be found in Table 1. The primary outcome targeted by the New Baby Programme is the parent (primarily the mother)-child relationship, assessed by measuring both maternal sensitivity and child attachment. In the event that the father is the primary carer, we will focus on his relationship with the child. We have chosen to use two measures as together they provide a better assessment of what is a complex, relational outcome. Attachment has been selected because of (i) the links between insecure and disorganised attachment and other parenting dimensions, such as maternal depression [44], substance misuse, neglect and maltreatment [45], and (ii) the centrality of attachment to healthy infant development, as indicated by the association between disorganised attachment and subsequent pathology, such as behaviour problems [46–50] and poor self-regulation [51]. Maternal sensitivity and responsiveness to infants’ needs is recognised as critical to the development of secure attachment [52, 53].

**Table 1:** Details and timing of data collection

Outcome	Measures	Average minutes to complete	Baseline, gestation 20 weeks	Infant 2 months	Infant 6 months	Infant 12 months
Attachment	Strange situation	20				X
Maternal sensitivity	CARE-Index	3				X
	Demographics	2	X	X (update)	X (update)	X (update)
Maternal depression	Edinburgh Postnatal Depression Scale (EPDS)	5		X	X	X
Infant questions	Brief questions on feeding and immunisations	.4	X (feeding plans)	X	X (feeding)	X
Lifestyle issues	Brief questions concerning drug use, smoking and alcohol use	1.2	X	X (update)		X (update)
Maternal quality of life	EuroQol (EQ-5D-5L)	5	X	X	X	X
Stress Antenatal stress Parenting stress	STAI-S-6	2	X	X	X	X
	NUPDQ	5	X			
	Parenting stress index, short form (PSI)	10	X	X		X
Parents’ sense of competence	Perceived parenting competence scale (PSOC)	5		X		X
Relationship violence	Brief questions	1	X			X
Social Networks	Medical Outcomes Study (MOS) social support survey short-form 36	5	X			X
Service use		3		X	X	X
Child development	Mullen Scales of Early Learning	15		X	X	X

In addition, a number of secondary outcomes have been selected to assess other aspects of parenting that are known to have a bearing on child development, such as parental mental health, smoking and substance use, neglect and maltreatment.

Measures have been selected primarily for their psychometric properties to support comparison with other studies, particularly Nurse-Family Partnership [54]. The NBP seeks to address similar issues and achieve comparable outcomes, albeit targeted at a different (primarily older) group of pregnant women, making direct comparisons of particular interest. The scheduling of data collection is designed to minimise the burden on participants, and is based on a tried and tested schedule used in a recent study of group Nurse-Family Partnership in England [54].

*Child attachment* will be measured using the Ainsworth Strange Situation Procedure (SSP) [55]. The SSP is a well-validated measure of attachment in infants aged 11–15 months.

*Maternal sensitivity* will be measured using the Infant CARE-Index (ICI). This observational measure uses a 3-min video recording of mother-child play and measures three aspects of maternal behaviour (sensitivity, covert and overt hostility, and responsiveness) and four aspects of infant behaviour (cooperativeness, compulsivity, difficultness, and passivity). These are highly correlated with attachment and also differentiate between abusing, neglecting, abusing and neglecting, marginally more treating and adequate dyads [56]. This means that they will allow us to assess the impact of the NBP on the participant’s parenting profile.

*Maternal depression* will be assessed using the Edinburgh Postnatal Depression Scale (EPDS) when the infant is 2, 6 and 12 months old [57]. This is a well-validated 12-item measure of postnatal depression with high reliability (0.88) and internal consistency (0.87), 86% sensitivity and 78% specificity. The questionnaire will be scored within 24 h of its administration, and any woman scoring above the recommended cut-off (indicating a risk of depression), or who responds affirmatively to the question asking about self-harm, will be brought to the attention of a healthcare professional so that appropriate support can be provided.

*Antenatal stress* will be measured at baseline using The Revised Prenatal Distress Questionnaire (NUPDQ). This is a 17-item measure of pregnancy-specific distress including physical discomforts, financial resources to care for children and pain during delivery [58].

*Postnatal anxiety* will be measured using the Strait Trait Anxiety Inventory Short Form-6 (STAI-6) at baseline and when the infant is 2, 6 and 12 months old [59]. The 6-item short-form of the STAI (STAI-6) has an acceptable reliability (0.82) and produces scores that are similar to those produced with the full-form 20-item STAI across participating groups manifesting normal and raised levels of anxiety. The STAI shows strong criterion, discriminant and predictive validity in perinatal populations [60].

*Parental stress* will be assessed using the Aberdeen Parenting Stress Index, Short Form (ASI-4-SF) at baseline and when the infant is 2 and 12 months old [61]. This is a well-validated 36-item measure of perceived stress in the parenting role. The 36 items are divided into three domains: parental distress, parent-child dysfunctional interaction, and difficult child, which combine to form a Total Stress Score. The ASI-4-SF has a robust test-retest reliability (*r* = 0.84) and internal consistency (*α* = 0.91). High scores on the PSI have been associated with abusive parenting, with some evidence that parenting stress is higher in women with five or more risk factors for child abuse.

Brief questions designed for an earlier study of group Family-Nurse Partnership [54] will be used to explore maternal smoking, alcohol and drug use, and relationship violence.

*Parent’s sense of competence* in the parenting role will be assessed using the Parenting Sense of Competence Scale (PSOC) when the infant is 2 and 12 months old [62]. The PSOC assesses parental competence on two dimensions: Satisfaction and Efficacy, established by factor analysis in a normative nonclinical sample, each with acceptable internal consistency (from 0.62 to 0.72). This is a 16-item Likert-style questionnaire (with a 6-point scale ranging from ‘strongly agree’ to ‘strongly disagree’).

*Maternal health-related quality of life* will be assessed using the EuroQol EQ-5D-5L. This is a standardised measure of health status developed by the EuroQol group that provides a simple, generic measure of health for clinical and economic appraisal [63]. The EQ-5D-5L consists of two pages. The first presents five dimensions of health (mobility, self-care, usual activities, pain/discomfort, anxiety/depression) and asks respondents to indicate their health status by ticking one of the following: no problems, slight problems, moderate problems, severe problems and extreme problems. The second presents a visual analogue scale that goes from 0 (worst health you can imagine) to 100 (best health you can imagine) and asks respondents to indicate with an ‘X’ where on the scale they see themselves. Responses can then be converted to a multi-attribute utility score by applying a UK tariff [64].

*Social support* will be assessed using the Medical Outcomes Study (MOS) Social Support Survey, a 20-item scale measuring four dimensions of support, established using a confirmatory factor analysis: emotional support, tangible support, positive interaction, and affection. Each has an internal consistency of 0.91 or above, and the measure provides a total support score (Cronbach’s alpha 0.97). Stability over time is high for each scale (ranging from 0.72 to 0.78) [65]. Parents’ use of local resources and services will be assessed using questionnaires originally designed for the group Nurse-Family Partnership study, along with brief questions about infant feeding and the take up of immunisation.

*Child development* will be assessed using the Mullen Scales of Early Learning when the child is 2, 6 and 12 months old [66]. The Mullen Scales comprise of five scales (Gross Motor, Visual Reception, Fine Motor, Expressive Language and Receptive Language) which are used for targeting strengths and weaknesses in children. The Mullen test is generally used for evaluating intellectual development and school readiness. Instrument assessments have supported various reliabilities. The median split-half internal consistency was above 0.80 for 3 of the subscales, but 0.79 and 0.75 for Visual Reception and Fine Motor. Test-retest intervals of 1 to 2 weeks and 1 to 24 months scored coefficients of 0.80 and 0.70, respectively.

Overall, response cards will be used for questions that provide a number of response options. All data will be collected during face-to-face interviews in the woman’s home, unless otherwise requested. In the event of respondents having low levels of literacy, the researcher will administer questions orally.

Outcome measures targeted by the intervention will be collected at baseline and 2, 6 and 12 months. Whilst the sample size in this pilot study is not sufficient to inform the power calculations that will be required for a phase 3 study, we will use the available data to estimate the standard deviation and interval estimates (i.e. 95% confidence intervals) of the outcome measures, as this will help inform those calculations (see NIHR guidance on feasibility studies).

### Study outcome measures

#### Primary outcomes—recruitment and retention rates

We will use the screening tools completed by midwives and the records of the senior nurse (see above) to estimate the number of women eligible for the trial and the percentage recruitment rate. Retention of participants will be determined via those for whom data are available at each data collection point. These data will address the feasibility (can we recruit and retain participants) and design (duration, number of recruitment sites required) of any future RCT.

#### Secondary outcomes—acceptability of intervention and study processes

Semi-structured interviews will be conducted with a subsample of mothers at 1 year postpartum, after the completion of other study data collection (thereby retaining the masking of the research team). These semi-structured interviews will explore their experiences of either the NBP or usual services, using a topic guide. Key issues will be the acceptability of the programme, its perceived helpfulness and their experiences of the trial process, including recruitment and follow-up. Women will also be asked about their experiences of randomisation and of data collection. Questions regarding data collection will encompass the frequency of data collection, the time required and the measures themselves. Particular attention will be given to the Strange Situation Procedure, which will be conducted at a specialist facility outside the home.

Women who decline to participate in the trial will also be asked if they are willing to take part in this qualitative study. If they give their consent, we will also conduct semi-structured interviews with them shortly after they decline to ascertain their reasons for not participating.

Because this is a pilot trial, we will conduct semi-structured interviews with key professionals involved in recruitment and referral, to explore their views on the feasibility of our strategy for recruitment, including the timing, materials and required resources. At the end of the study, we will also conduct semi-structured interviews with health visitors delivering a service in both arms of the trial and other key professionals (for example, supervisors, managers, consultants).

#### Researcher blinding

The study has been designed to maximise the chances of the research team (other than the person responsible for data entry) to remain blind to the allocation status of participants, particularly with regard to those conducting data collection. Women have been asked not to disclose their allocation status to those collecting data, and the latter remind women of this prior to each visit. Outcome assessors are also asked to record any indication given of the allocation status of participants during each contact with participants. These will be reported and taken into account as an indication of potential bias.

### Economic evaluation

Economic data, including resource use and unit costs, will be collected in parallel with the trial. Resource use information will be derived at the individual level from parental self-report on use of local resources and health and social services. A resource measurement tool designed and standardised for the group Nurse-Family Partnership study will be used. Health visitors delivering the intervention will record the time spent and activities undertaken in support of the parent-infant dyad.

NBP costs will be calculated using a standard micro-costing (bottom-up) approach and will be based on health visitor salaries plus on-costs (employers’ national insurance and superannuation contributions) and appropriate capital, administration and training costs. Nationally applicable unit costs will be applied to all community health and social care contacts, derived from the Personal Social Services Research Unit (PSSRU), and medications, derived from the British National Formulary. Costs for NHS hospital contacts will be taken from NHS reference costs.

### Data management

All research staff will receive training on data collection and other protocols, and the trial manager will monitor the quality of data collection by accompanying each researcher on some home visits during the study.

Data will be managed in accordance with Queen’s Standard Operating Procedure (SOP) for the management of data, in particular, electronic data. Available through *Queen’s On-line*, this SOP details how data are to be captured, in particular, with the informed consent of the research participant and giving details as to the purpose for which the information is to be used, the period of time it is to be retained and to whom it is likely to be disclosed. It is ICH GCP 1996 compliant. A study database will be established that is designed to ensure completeness, accuracy, reliability and consistency.

To ensure quality, all data will be double entered and a percentage of completed forms will be reviewed by the person responsible for data entry. Prior to data entry, each case report form will be checked for incomplete or missing information and any inconsistencies checked with the relevant researcher. A record will be kept of all queries raised and the response received.

Efforts will be made to obtain outcome measures from all participants enrolled in the trial, including any who withdraw from the study after randomisation.

### Data analyses

#### Analysis of process and qualitative data

Characteristics of participants recruited to each arm will be collected at baseline and used to assess patterns of attrition and the extent to which attrition rates appear to be associated with either arm of the trial. Data collected will include demographic data (e.g. age, ethnicity, marital status) and social complexities present. We will record details of women who decline to participate and use these data and those data available on NIMATS/CHS of those approached and those recruited, using age, parity and reasons for social complexity (where recorded, e.g. domestic violence, substance misuse, mental health problems).

The uptake rate of women agreeing to the intervention will be based on an assessment of the ratio of women randomised to the intervention group who then meet with the NBP health visitor on at least one occasion, relative to those who agree to take part in the trial and are randomised to NBP but do not attend the first (or any subsequent) meeting. The attrition rate will be calculated on the basis of the percentage of women who drop out relative to those who continue in either randomised group, together with those who subsequently decline to meet with their health visitor, irrespective of whether or not they continue to meet with the study researchers. Analyses of retention and compliance rate will inform estimates of likelihood of adverse events (e.g. withdrawal from the study), which will be important in designing the phase 3 RCT.

Whilst the sample size in this pilot study is not sufficient to inform the power calculations that will be required for a phase 3 study, we will use the available data to estimate variability of the outcomes, as this will help inform those calculations (see NIHR guidance on feasibility studies). To this aim, we will report the statistics obtained, interval estimates (e.g. 95% confidence intervals) and other sample statistics that may help inform power calculations.

Treatment integrity will be assessed by means of the records completed by health visitors at every visit/contact, plus data from the semi-structured interviews conducted at the end of the study, with nurses and women participants.

All semi-structured interviews will, with the permission of participants, be digitally recorded and later transcribed and analysed thematically, using NVivo (a computer-aided qualitative data analyses package).

#### Analysis of outcome data

Demographic data will be presented for all participants enrolled in the trial. No significance tests will be conducted to explore baseline differences. For continuous variables, we will report the mean, standard deviation, median, range and number of observations. Categorical variables will be reported in numbers and percentages.

The unit of analysis will be the individual participant. All primary analyses will be conducted according to intention-to-treat principles, irrespective of participants’ exposure to the programme, e.g. irrespective of how many home visits were achieved. The research team will endeavour to secure outcome measures for all participants, including those that move out of the area, or who subsequently decline to accept the programme. Examination of missing data (both case and item) will be undertaken on outcome measures and covariates, and particular attention will be paid to differential attrition and the possible reasons for this.

In the event of marked differential attrition or large amounts of missing data, we will consider methods such as inverse probability weighting to account for this. Sensitivity analyses will be conducted for all primary outcomes. Minimally, these will explore the potential impact of outliers and of missing data and the impact on the results of excluding participants who do not receive the programme, for whatever reason (because intention-to-treat analyses may underestimate the potential efficacy of an intervention).

We will use multiple regression modelling, with appropriate generalised linear models (GLMs) to explore the impact of the intervention, adjusting for the outcome measures at baseline. A statistical analysis plan will be developed in advance of the completion of data collection. This will include a small number of secondary analyses that either test emerging hypotheses (for example, concerning the programme’s impact on particular subgroups of mothers) or explore the effect of dose or compliance. We will conduct these analyses as an aid to estimating interval estimates (e.g. 95% confidence intervals) and variability in outcomes, in particular, to estimate between- and within-group variability: this information will be useful in planning the phase 3 RCT.

We do not anticipate undertaking any subgroup analyses in this pilot trial. We will, however, use the pilot trial to assess the feasibility of recruiting different subgroups that might be particularly important for the analyses of the main trial. In the main trial, we would anticipate using subgroup analyses to explore the moderating effects of factors such as nature of social complexity (e.g. presence or absence of substance misuse; presence or absence of history of maltreatment) or family status (e.g. primaparous, multiparous; maternal age).

#### Analyses of economic data

The economic evaluation will explore the incremental cost-effectiveness of the NBP compared to Management as Usual (MAU) at the 12-month follow-up. Firstly, a total cost per case per parent-infant dyad will be calculated to allow for a mean cost per case per trial arm for the intervention and control group, with rates of significance of difference between arms. Secondly, cost-effectiveness will be measured in terms of health-related quality of life (EQ-5D-5L) to determine incremental costs per quality-adjusted life year. Non-parametric bootstrapping will be used to generate a distribution of mean costs and effects for the two trial arms and develop confidence intervals around the incremental cost-effectiveness ratio. This process will also generate acceptability curves to illustrate the uncertainty associated with the estimate of costs and effects combined and estimates of affordability given potentially different decision-maker cost thresholds. The probability that the NBP could be more cost-effective compared with MAU for a range of maximum monetary values will then be explored.

In the main trial, longer-term implications of NBP will be explored with data from the main trial extrapolated and supplemented with data from the literature using decision analytic modelling techniques. The pilot trial will assist in identifying potential impacts of NBP for mothers who experience social complexity and their infants.

## Discussion

### Importance of the study

There is a need for effective antenatal and postnatal provision for mothers whose circumstances are socially complex. To date, the focus in the UK has been primarily on young, socio-economically disadvantaged women who are expecting their first child. The Nurse-Family Partnership programme developed by David Olds and his colleagues in the USA enjoys the strongest evidence base to date and has been trialled in the UK (as the Family Nurse Partnership, FNP) in both its original mode of delivery (one home visitor to one mother, with visits taking place in the mother’s home) and in a group-based version in which mothers meet in groups of around eight, led antenatally by a midwife and health visitor, and postnatally by a health visitor. Both models are being evaluated [54, 67]. The NFP was developed in a context without universal services available to pregnant women and mothers, free at the point of delivery and, with some exceptions in its group format, has not been available to multiparous or older pregnant women. In contrast, the New Baby Programme is designed to augment the UK’s universal service and to meet the needs of a significant group of women who are ineligible for FNP. Before seeking funding for a full trial, it is important to ensure that the research processes and procedures are feasible and acceptable and indicate whether or not a full trial is justified. This pilot trial is designed to address both issues.

### Determining the feasibility of a randomised controlled trial

Experimental evaluations of complex social interventions with hard to reach groups are challenging on a number of fronts. This pilot study will help us to identify optimum recruitment procedures, the likely time it will take to recruit a sufficiently powered sample size (in conjunction with other studies using the primary outcome measures), what the barriers and facilitators of study participation might be (e.g. does a £20 voucher assist with retention) and whether the intervention is acceptable to participants and relevant professional staff.

In the main trial for which this study is a pilot, the optimum measures for the primary outcome include two measures of a child-parent interaction: the Infant CARE-Index [68] and the Strange Situation Procedure [55]. Whilst the first can be undertaken in the family home and comprises only a 3-min video recording of mother-child play, the Strange Situation Procedure is an eight-step laboratory-based procedure which will require parents to attend somewhere equipped to conduct it. The sequence of steps, each of which lasts 3 min, is as follows: (a) parent and child are introduced to the room, and the procedure is briefly explained to the parent; (b) the parent and child are left alone; (c) a stranger joins the parent and child; (d) *first separation* (parent leaves the room); (e) parent comes back and the stranger leaves—*first reunion*; (f) *second separation* (parent leaves the room); (g) stranger comes in; and (h) parent comes back into the room—*second reunion*. Although participants are made aware of these expectations prior to agreeing to participate in the study, an important aspect of this pilot is identifying the extent to which they will be willing to collaborate with the research team in providing these data.

### Risks and benefits for participants

Whilst some expectant mothers will be allocated not to receive the programme under investigation, it is important to determine whether NBP is likely to have an identifiable impact. Up to this point, the evaluation of the NPP has been based on the perceptions of key stakeholders, including participants [69], and on the acceptability and impact of the NPP on their lives, but without a control group who are receiving regular services. It would be a greater risk to offer a programme without establishing that it has specific benefits.

There will be benefits for the families involved whether they receive the programme or not. They will experience four research visits (as well as the Strange Situation Procedure), with a small monetary recompense, and will be able to talk about their early parenting, and experience in other studies suggests this is valued by families. Any family thought to be in need of referral to specialist services will be urged by the research team to do this, through their GP, and will be supported by the researchers.

Information provided will be kept strictly confidential. However, in line with usual practice, if a researcher identifies any concerns about a participant, their unborn baby, or a child who may be at risk, they will be required to share this information with the relevant authorities, having first informed the participant. Information to this effect is included in the Patient Information Sheet.

### Dissemination

We will make public the results of the study via an open access journal publication, a final technical report and briefing for the funders of the study and a plain English summary which we will send to all participants.

### Trial status

**Fig. 2:**
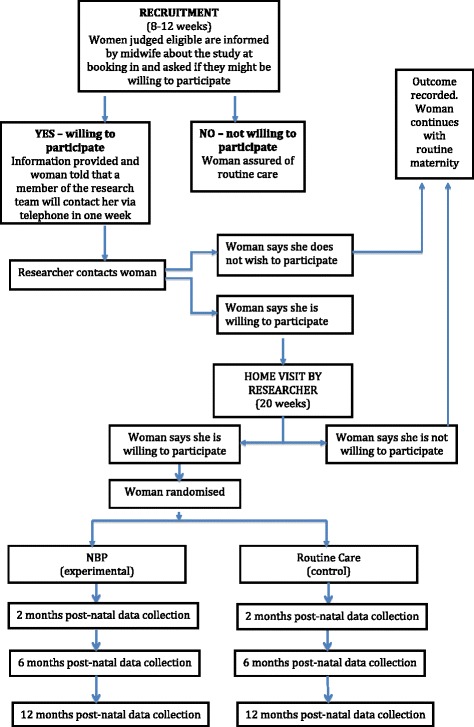
Flow of participants

The recruitment of potential participants started in April 2016. The trial will be completed in August 2018. Figure 2 provides the details of the timeline for each participant, extending from their booking-in visit at the antenatal clinic (usually at around 8–18 weeks of gestation) through to the end of their participation in the study, when their children are 12 months old. The duration of each participant’s involvement in the study will vary with their gestation at booking but will be in the range from 80 to 84 weeks. The intervention group will receive the NBP for 72 weeks on average (from 16 weeks of gestation to infant age 12 months).
